# (De-)centralized health care delivery, surgical outcome, and psychosocial health of transgender and gender-diverse people undergoing vaginoplasty: results of a retrospective, single-center study

**DOI:** 10.1007/s00345-023-04348-5

**Published:** 2023-03-24

**Authors:** Andreas Koehler, Bernhard Strauß, Peer Briken, Margit Fisch, Silke Riechardt, Timo O. Nieder

**Affiliations:** 1 Institute for Sex Research, Sexual Medicine, and Forensic Psychiatry, University Medical Center Hamburg-Eppendorf, Hamburg, Germany; 2 Interdisciplinary Transgender Health Care Center, University Medical Center Hamburg-Eppendorf, Hamburg, Germany; 3 Institute of Psychosocial Medicine, Psychotherapy, and Psycho-Oncology, University Hospital Jena, Jena, Germany; 4 Department for Urology, University Medical Center Hamburg-Eppendorf, Hamburg, Germany

**Keywords:** Transgender health, Gender-affirming surgery, Surgical outcome, Health care delivery, Health services research

## Abstract

**Purpose:**

Previous research on genital gender-affirming surgery lacked to build a framework that took various surrounding factors into account. E.g., transgender health care services are delivered in both centralized (by one interdisciplinary institution) and decentralized settings (by different medical institutions spread over several locations). The present study investigated the effects of different structural and clinical aspects of gender-affirming genital surgery on psychosocial outcomes.

**Methods:**

We surveyed former transgender and gender-diverse people who completed a vaginoplasty between 2014 and 2018. 45 participants were included in the study. We calculated hierarchical linear regression analyses to assess the relationship between psychosocial outcome measures (gender congruence, mental health, quality of life) and different aspects of gender-affirming genital surgery (e.g., setting of service delivery). To address shortcomings regarding the small sample size, we applied a rigorous statistical approach (e.g., Bonferroni correction) to ensure that we only identify predictors that are actually related to the outcomes.

**Results:**

A non-responder analysis revealed no systematic bias in the recruitment procedure. Treatment satisfaction was a significant predictor for gender congruence. Moreover, we found the setting of service delivery (centralized, decentralized) to predict psychological health and the physical health dimension of quality of life. The effect sizes of our models were moderate to high, and models explained up to 26% of the total variance with a power up to 0.83.

**Conclusion:**

The present study is an exploratory attempt into the manifold relationships between treatment-related factors (e.g., aesthetic outcome), the setting of service delivery, and their effects on gender-affirming genital surgery.

**Supplementary Information:**

The online version contains supplementary material available at 10.1007/s00345-023-04348-5.

## Introduction

Transgender and gender-diverse people experience their gender as incongruent to the sex they were assigned to at birth (ICD-11: Gender Incongruence). This can lead to clinically relevant distress referred to as gender dysphoria (DSM-5). About 80% of transgender and gender-diverse people identify as the ‘opposite’ gender (male or female), whereas 20% identify as non-binary [1]. This includes genders that oscillate between the male and female parts (e.g., genderfluid), are situated beyond the gender binary (e.g., genderqueer), or reject the gender binary at all (e.g., agender).

**Fig. 1:**
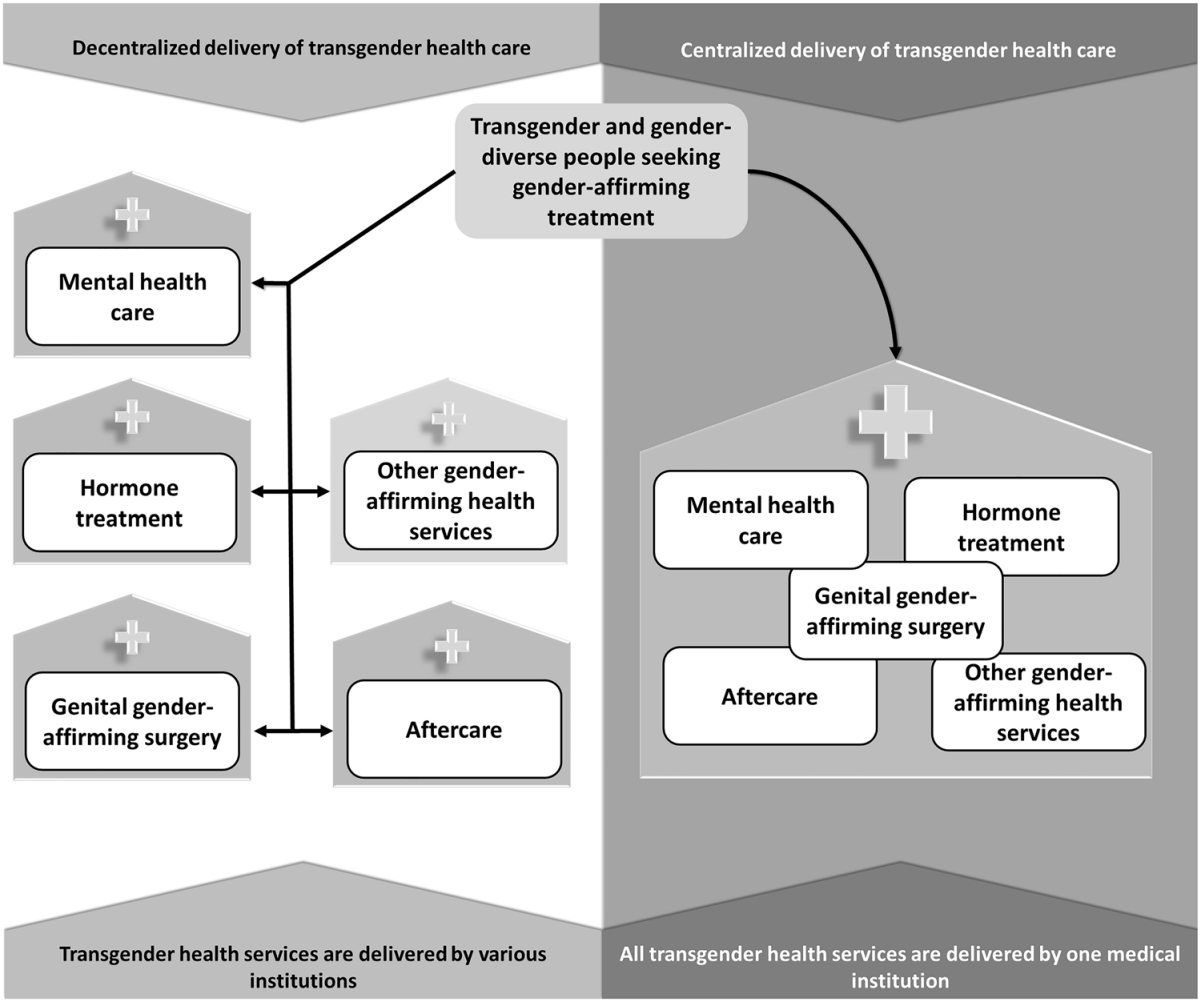
Centralized and decentralized delivery of transgender health services

For transgender and gender-diverse people, gender dysphoria often leads to the desire to modify primary and secondary sex characteristics and live according to their experienced gender. Therefore, they might assess a variety of medical interventions, all of which can be very effective in the process of transitioning into the experienced gender [2]. Common medical interventions are hormonal therapy and gender-affirming genital surgery. Moreover, breast removal or augmentation, phono and facial surgery, and further procedures like speech therapy and hair removal treatment are applied [2]. The World Professional Association for Transgender Health published the current treatment guidelines in their 8th version of the standards of care (SoC8) [3]. The goal of the SoC8 is both to promote inclusive, evidence-based health care for all gender identities and ensure access to transgender health care. Currently, gender-affirming procedures are both delivered within centralized settings, where a specialized, interdisciplinary center provides all interventions a person wants to undergo, and decentralized services, where procedures are provided by different institutions (Fig. 1) [4, 5]. It has been found that the (de-)centralized delivery of transgender health services could affect the quality and the tailored provision of transgender health services [6].

For transgender and gender-diverse people assigned female at birth, hysterectomy, oophorectomy, metoidioplasty, and phalloplasty are common genital gender-affirming procedures [2, 7]. For transgender and gender-diverse people assigned male at birth, orchiectomy, penectomy, and vaginoplasty are established surgical procedures for genital gender affirmation [2]. Several studies found that gender-affirming genital surgery can significantly lower gender dysphoria, enhance the quality of life and mental health, and improve the sexual health of transgender and gender-diverse people [8, 9]. Regarding complication and dissatisfying aesthetical results, stenosis of the neo-meatus has been found as the primary complication (11% of cases) is a recent systematic review [10]. The same was true for masculinizing gender-affirming genital surgery [11]. However, for all studies included in these reviews, the quality of evidence was rated low by the authors [8, 9, 11, 12]. Moreover, the authors criticized the lack of comparability due to the use of both different and partly unstandardized outcome measures and treatment protocols. The reviews highlight the need for patient-reported outcome measures to improve evaluating gender-affirming surgery from the patient’s perspective [8, 9, 11, 12]. Additionally, previous research primarily focused on relationships between single predictors and both surgical and psychosocial outcomes. However, it failed to develop a holistic framework of the various factors that might influence the outcome of gender-affirming genital surgery.

The present study investigates the effects of different structural and clinical aspects of gender-affirming genital surgery (setting of service delivery, treatment satisfaction, aesthetic outcome, functional outcome) on psychosocial outcomes (gender dysphoria, mental health, quality of life) using standardized measures. We aim to address criticism of previous research and contribute to a more nuanced understanding of the various aspects potentially influencing the psychosocial outcome of gender-affirming genital surgery.

## Methods

### Study design

This retrospective study was conducted by the Institute for Sex Research, Sexual Medicine and Forensic Psychiatry and the Department for Urology, both located at the University Medical Center Hamburg-Eppendorf (UKE), Germany. Both are part of the Interdisciplinary Transgender Health Care Center Hamburg. It was performed of a single surgeon’s experience (SR) and was part of a larger research project on client-centered health care for transgender and gender-diverse people [13]. The study received ethical approval from the Chamber of Psychotherapists Hamburg Ethics Committee (10/2018-PTK-HH).

### Participants

Participants had to be at least 16 years of age and underwent a two-step vaginoplasty using penile inversion technique to be eligible for study participation. All former patients who completed a vaginoplasty between 2014 and 2018 were invited to participate.

### Participant recruitment

The data collection took place between January and March 2020. 116 former patients were contacted and asked to participate in the study. 45 responded to our inquiry and were included in the study (response rate of 38.8%). Written consent was obtained from all participants. To assess a systematic bias in the recruitment procedure, a non-responder analysis was performed by comparing age and the size of place of residence between participants and non-participants.

### Measures

The present analysis investigated data on the aesthetic and functional outcome of vaginoplasty, satisfaction with the treatment, gender congruence, mental health, and quality of life. When participants received counseling and maybe other treatments within the Interdisciplinary Transgender Health Care Center Hamburg, they were considered receiving care in a centralized healthcare delivery setting. Those who only underwent vaginoplasty at the Department for Urology were categorized as accessing transgender health care decentralized (Fig. 1). We used the Transgender Congruence Scale, Brief Symptom Inventory-18, WHOQOL-BREF, Female Genital Self Image Scale (FGSIS), Female Sexual Functioning Index (FSFI), and an adapted Short Questionnaire for Self-Evaluation of Vaginoplasty (SQSV; see supplementary material for detailed references) as patient-reported outcome measures.

### Data analyses

The statistical analyses were conducted using SPSS 27.0. Missing data were deleted pairwise. The sample characteristics and outcomes of the questionnaires were reported descriptively. To illustrate the progress in transgender-related treatment, the Individuals Treatment Progress Score [1] was calculated. The Mann–Whitney *U* test was calculated to assess differences between participants accessing transgender health care in centralized and decentralized delivery settings. We calculated hierarchical linear regression analyses to assess the relationship between psychosocial outcome measures (gender congruence, mental health, quality of life) and different aspects of gender-affirming genital surgery (treatment satisfaction, aesthetic outcome, functional outcome, setting of health care delivery, details see supplementary material).

Normal distribution of data and residuals were examined using histograms and Q–Q plots. The assumption of the independence of the observations was assessed using the Durbin–Watson statistic. Multicollinearity was excluded by inspecting correlation coefficients and tolerance/VIF (Variance inflation factor) values. Heteroscedasticity was assessed by examining scatterplots of predicted residuals. Using G*Power [14], we calculated the minimum sample size necessary to find a significant effect a priori. Due to the lack of high-quality evidence [8, 9, 11, 12], we assumed a large effect of our predictors on the psychosocial outcome based on clinical experience. Therefore, multiple regression analysis with five predictors and a power of 0.80 needs a sample size of at least *N* = 43 to determine a large effect (*f*^2^ = 0.35). We used Cohen’s *f*^2^ as a post hoc measure of the effect size of the regression models. According to Cohen’s conventions, *f*^2^ = 0.02 indicates a small effect, *f*^2^ = 0.15 indicates a medium effect, and *f*^2^ = 0.35 indicates a large effect [15]. We also used G*Power [14] to perform a post hoc calculation of the achieved power of our models. For the non-responder analysis, a *t* test for independent samples and a chi-square test were performed to compare the age and population of the place of residence between participants and non-participants. All analyses were performed with an alpha level of 0.05, and—to handle the problem of multiple comparisons—a Bonferroni-corrected [16] alpha level of 0.01 (0.05 divided by the 5 predictors of the hierarchical regression analyses).

## Results

The mean age of participants was 43.4 ± 15.6 (range 19.0–69.0). On average, the participants were surveyed 2.6 ± 1.1 years after vaginoplasty. Details on demographic characteristics are listed in Table 1. Gender- and treatment-related information are given in Table S1 (supplementary material).

**Table 1:** Demographic characteristics

	Total sample (Mdn, no., %)	Centralized health care delivery (Mdn, no., %)	Decentralized health care delivery (Mdn, no., %)	Statistics
*N*	45 (100.0)	24 (53.3)	21 (46.7)	
Age, mean (SD)	43.4 (15.6)	51.5 (mean rank = 26.48)	36.0 (mean rank = 19.02)	U = 335.500; *p* = 0.057
Country of birth				
Germany	38 (84.4)	20 (83.3)	18 (85.7)	
Other European countries	2 (4.4)	1 (4.2)	1 (4.8)	
Non-European countries	2 (4.4)	0 (0.0)	2 (9.5)	
Cannot or do not wish to answer this question	3 (6.6)	3 (12.5)	0 (0.0)	
Population of the place of residence				
< 5000	5 (11.1)	2 (8.3)	3 (14.3)	
5000–20,000	6 (13.3)	2 (8.3)	4 (19.0)	
20,000–100,000	6 (13.3)	2 (8.3)	4 (19.0)	
100,000–1,000,000	3 (6.6)	1 (4.2)	2 (9.5)	
> 1,000,000	17 (37.8)	11 (45.8)	6 (28.6)	
I do not know	3 (6.6)	2 (8.3)	1 (4.8)	
Cannot or do not wish to answer this question	5 (11.1)	4 (4.2)	1 (4.8)	
Marital status				
Single	19 (42.2)	11 (45.8)	8 (38.1)	
In a relationship	9 (20.0)	2 (8.3)	7 (33.3)	
Married, living together	5 (11.1)	4 (16.6)	1 (4.8)	
Married, living separately	2 (4.4)	1 (4.2)	1 (4.8)	
Registered partnership, living together	2 (4.4)	1 (4.2)	1 (4.8)	
Divorced	3 (6.6)	1 (4.2)	2 (9.5)	
Widowed	2 (4.4)	1 (4.2)	1 (4.8)	
Cannot or do not wish to answer this question	3 (6.6)	3 (12.5)	0 (0.0)	
Education				
Low	8 (17.8)	4 (16.6)	4 (19.0)	
Middle	12 (26.7)	6 (25.0)	6 (28.6)	
High	20 (44.4)	10 (41.7)	10 (47.6)	
Cannot or do not wish to answer this question	5 (11.1)	4 (16.6)	1 (4.8)	
Employment				
Full-time	17 (37.8)	12 (50.0)	5 (23.8)	
Part-time	5 (11.1)	2 (8.3)	3 (14.3)	
Mini-job (i.e., individual earnings < 400€/month)	4 (8.8)	0 (0.0)	4 (19.0)	
Unemployed	6 (13.3)	3 (12.5)	3 (14.3)	
Retired	4 (8.8)	2 (8.3)	2 (9.5)	
Cannot or do not wish to answer this question	9 (20.0)	5 (20.8)	4 (19.0)	
Occupational status				
Student	2 (4.4)	1 (4.2)	1 (4.8)	
Vocational training	4 (8.8)	1 (4.2)	3 (14.3)	
Unskilled worker	2 (4.4)	0 (0.0)	2 (9.5)	
Operative	1 (2.2)	0 (0.0)	1 (4.8)	
Employee	22 (48.9)	14 (58.3)	8 (38.1)	
Civil servant	1 (2.2)	0 (0.0)	1 (4.8)	
Self-employed	3 (6.6)	0 (0.0)	3 (14.3)	
Cannot or do not wish to answer this question	10 (22.2)	8 (33.3)	2 (9.5)	

Results on the aesthetic outcome of the vaginoplasty (FGSIS), the functional outcome of the vaginoplasty (FSFI), the SQSV on both aesthetic and functional outcomes, and free text responses on urinary problems after vaginoplasty are listed in the supplementary material (Tables S2, S3, S4, S5).

The hierarchical multiple regression analysis (Table 2) for gender congruence indicated treatment satisfaction as the only significant predictor. Our final model explained 23% of the total variance. The effect of this model is considered as medium (*f*^2^ = 0.30). The final model achieved a power of 0.76. Regarding the final model on psychological distress (Table 2), the setting of health care delivery was the only significant predictor. The final model explained 26% of the total variance. The effect of the entire model was large (*f*^2^ = 0.35). The final model achieved a power of 0.83. For the final model regarding the physical health dimension of quality of life (Table 2), the setting of health care delivery, again, was the only significant predictor. The final model explained 21% of the total variance. The effect of the entire model was medium (*f*^2^ = 0.27). The final model achieved a power of 0.70. None of the included variables had a significant predictive power for the other quality of life dimension*s* (Table 2). The Durbin–Watson statistics indicated the independence of observations for all models. Correlation coefficients and tolerance/VIF values did not indicate multicollinearity in all models. Also, we found no evidence of heteroscedasticity in all models by examining scatterplots of predicted residuals.

**Table 2:** Multiple regression analysis for gender congruence, psychological distress, and quality of life

	Gender congruence [20]							
	Coefficients of the final model (step 4)				Model summary			
	*B* ± SE	CI	*β*	*p*	Model	*R*^2^	Δ*R*^2^	Sign. Δ*F*
Constant	3.35 ± 0.67	2.12–4.85		0.000	1	0.13	0.13	0.029
Satisfaction	0.12 ± 0.05	0.15− 0.23	0.37	0.026*	2	0.20	0.08	0.085
FGSIS	0.03 ± 0.02	− 0.02− 0.08	0.23	0.188	3	0.22	0.02	0.646
FSFI	0.01 ± 0.01	− 0.01− 0.02	0.09	0.589	4	0.23	0.00	0.831
Urinary problems	− 0.22 ± 0.26	− 0.75− 0.31	− 0.14	0.401				
Delivery setting	− 0.03 ± 0.14	− 0.32− 0.26	− 0.04	0.831				

	Psychological distress [21]							
	Coefficients of the final model (step 4)				Model summary			
	*B* ± SE	CI	*β*	*p*	Model	*R*^2^	Δ*R*^2^	Sign. Δ*F*
Constant	22.04 ± 8.02	5.68–38.40		0.010	1	0.03	0.03	0.314
Satisfaction	− 0.90 ± 0.62	− 2.17− 0.37	− 0.23	0.158	2	0.05	0.02	0.412
FGSIS	− 0.30 ± 0.27	− 0.85− 0.24	− 0.19	0.267	3	0.08	0.03	0.615
FSFI	− 0.03 ± 0.10	− 0.23− 0.17	− 0.05	0.762	4	0.26	0.16	0.010
Urinary problems	− 4.16 ± 3.19	− 10.52–2.20	− 0.21	0.192				
Delivery setting	− 4.64 ± 1.70	− 8.10—− 1.17	− 44	0.009*^;†^				

	Quality of life (physical health) [25]							
	Coefficients of the final model (step 4)				Model summary			
	*B* ± SE	CI	*β*	*p*	Model	*R*^2^	Δ*R*^2^	Sign. Δ*F*
Constant	11.60 ± 2.66	6.16–17.03		0.000	1	0.02	0.02	0.456
Satisfaction	− 0.07 ± 0.21	− 0.49− 0.35	− 0.05	0.745	2	0.03	0.01	0.484
FGSIS	0.08 ± 0.09	− 0.11− 0.26	0.15	0.404	3	0.06	0.03	0.633
FSFI	0.00 ± 0.03	− 0.06− 0.07	0.02	0.993	4	0.21	0.15	0.022
Urinary problems	− 0.74 ± 1.04	− 2.86–1.37	− 0.12	0.478				
Delivery setting	1.36 ± 0.57	0.21–2.51	0.40	0.022*				

	Quality of life (psychological) [25]							
	Coefficients of the final model (step 4)				Model summary			
	*B* ± SE	CI	*β*	*p*	Model	*R*^2^	Δ*R*^2^	Sign. ΔF
Constant	10.72 ± 3.06	4.47–16.97		0.001	1	0.00	0.00	0.909
Satisfaction	0.05 ± 0.24	− 0.43− 0.54	0.04	0.827	2	0.10	0.10	0.061
FGSIS	0.17 ± 0.10	− 0.04− 0.38	0.29	0.106	3	0.12	0.02	0.677
FSFI	0.03 ± 0.04	− 0.04− 0.11	0.15	0.397	4	0.19	0.07	0.127
Urinary problems	− 0.60 ± 1.12	− 3.03–1.83	− 0.08	0.618				
Delivery setting	1.02 ± 0.65	− 0.31–2.34	0.26	0.127				

	Quality of life (social relationships) [25]							
	Coefficients of the final model (step 4)				Model summary			
	*B* ± SE	CI	*β*	*p*	Model	*R*^2^	Δ*R*^2^	Sign. Δ*F*
Constant	10.78 ± 5.35	− 0.14–21.67		0.053	1	0.01	0.01	0.631
Satisfaction	− 0.19 ± 0.42	− 1.04− 0.65	− 0.08	0.645	2	0.06	0.05	0.172
FGSIS	0.15 ± 0.18	− 0.21− 0.52	0.16	0.397	3	0.12	0.06	0.383
FSFI	0.09 ± 0.06	− 0.04− 0.52	0.25	0.182	4	0.12	0.00	0.979
Urinary problems	− 0.12 ± 2.08	− 4.37–4.12	− 0.01	0.953				
Delivery setting	− 0.03 ± 1.13	− 2.28–2.34	0.01	0.979				

	Quality of live (environment) [25]							
	Coefficients of the final model (step 4)				Model summary			
	*B* ± SE	CI	*β*	*p*	Model	*R*^2^	Δ*R*^2^	Sign. Δ*F*
Constant	11.53 ± 3.72	3.94–19.12		0.004	1	0.00	0.00	0.735
Satisfaction	− 0.00 ± 0.29	− 0.59− 0.59	− 0.00	0.996	2	0.10	0.10	0.065
FGSIS	0.18 ± 0.12	− 0.07− 0.44	0.25	0.151	3	0.17	0.07	0.291
FSFI	0.07 ± 0.05	− 0.02− 0.16	0.26	0.126	4	0.25	0.08	0.083
Urinary problems	− 1.32 ± 1.45	− 4.27–1.63	− 0.15	0.368				
Delivery setting	1.41 ± 0.79	− 0.19–3.02	0.29	0.083				

*Statistically significant on an alpha level of 0.05, ^†^statistically significant on a Bonferroni-corrected alpha level of 0.01

A non-responder analysis revealed no significant differences between participants and non-participants with regard to age (*t*(115) = 0.166, *p* = 0.868). Moreover, a chi-square-test revealed no significant differences concerning the size of the place of residence between participants and non-participants (*χ*^2^(4, *N* = 116) = 0.810, *p* = 0.937).

## Discussion

The present study investigated the effects of various structural and clinical aspects of gender-affirming genital surgery (setting of service delivery, treatment satisfaction, aesthetic outcome, functional outcome) on psychosocial outcomes (gender dysphoria, mental health, quality of life).

Regarding demographical variables, e.g., age, education, our sample was comparable to those from prior research [1, 17, 18]. However, we found the group undergoing gender-affirming transgender health care in a decentralized setting to be slightly younger (median age 36.00 vs. 51.50; n.s). Most of our participants reported a binary female gender. Only 6.8% were non-binary. This is in line with prior research that found non-binary transgender and gender-diverse people were less likely to undergo gender-affirming genital surgery [1, 19]. As non-binary transgender and gender-diverse people often reject a distinct allocation to one gender, it seems reasonable that surgical procedures which promote such an assignment are less likely to be undergone. Our participants reported high satisfaction with the aesthetic results of vaginoplasty. The aesthetic satisfaction after vaginoplasty was comparable to satisfaction in cisgender samples [20] and was also in line with results from prior research investigating transgender and gender-diverse people after gender-affirming genital surgery [17]. On the other hand, the functional outcome of vaginoplasty was poor. All of our participants qualified as sexually dysfunctional according to the cut-off of the FSFI. Their functional satisfaction after vaginoplasty was comparable with cisgender women with sexual problems [21]. However, it is important to note that 44.4% of our sample reported no sexual activity in the last four weeks. They were scored as 0 on the FSFI and, therefore, considered as having a poor functional outcome. Therefore, these data need to be interpreted with care. 11.1% of our participants had urinary problems after the vaginoplasty, which is lower than reported in systematic reviews of prior research [12]. Also, 20.0% described their vagina as not deep enough for penetrative sexual intercourse, which might have contributed to the low scores on the FSFI. These results reflect the common challenge that gender-affirming genital surgery is still often associated with postsurgical functional problems [12]. An additional in-depth discussion of the univariate results of our study can be found in the supplementary material.

Regarding the relationship between structural and clinical aspects of vaginoplasty and psychosocial outcomes (gender incongruence, psychological distress, quality of life), we found the overall satisfaction with the treatment and the setting of health care delivery (centralized, decentralized) to be significant predictors. Higher overall satisfaction with the procedure was associated with higher gender congruence. Rather than focusing on individual gender congruence, most prior research assessed gender incongruence or gender dysphoria using measurement tools based on stereotyped assumptions concerning sex and gender [22]. We consider the Transgender Congruence Scale a more inclusive measure of gender focusing on the individual feeling about one’s body, independent of anatomical features that are considered sex/gender-specific. Even though the instrument is a well-established measurement tool in transgender research, it has been chiefly used in non-clinical samples (e.g., [23, 24]). In a clinical setting, Owen-Smith and colleagues found a positive association between gender-affirming procedures and enhanced gender congruence [25]. Isung et al. focused on craniofacial surgery and found a significant postsurgical improvement in gender congruence [26]. The results of our study support this prior research. However, treatment satisfaction as a predictor did not survive Bonferroni correction, and the regression model on gender congruence only achieved a power of 0.76. Therefore, future research needs to investigate this relationship in larger samples.

The setting of health care delivery was found as a predictor for psychological distress and the physical health dimension of quality of life. Accessing gender-affirming transgender health care in a centralized setting was associated with better physical health and lower psychological distress. Prior research revealed the advantages of centralized transgender health care delivery as a comprehensive, patient-centered model of services, providing standardized health services by health care professionals with specific training [6]. Positive outcomes of gender-affirming interventions delivered in centralized settings have been described in various studies [27–30]. This could have contributed to better physical and mental health of participants accessing transgender health care in centralized settings in the present study, too. However, only the result on psychological distress survived Bonferroni correction and had sufficient power of 0.83 in the final model. Therefore, future research should systematically address the setting of health care delivery to get a more nuanced insight into its role in high-quality transgender health care.

Despite the standardized approach used by the present study, the results cannot be generalized. The number of participants was small, which only allowed us to find large effects with sufficient power. To investigate medium or small effects, higher powered studies are necessary. However, we addressed these shortcomings by applying a rigorous statistical approach (e.g., Bonferroni correction) to ensure that we only identify those predictors that are actually related to the outcomes. Also, the protocol needs to be transferred to research other gender-affirming procedures (e.g., phalloplasty). We used a retrospective approach to study transgender and gender-diverse people that were undergoing vaginoplasty. For a more pronounced view into the effects of the surgical procedures on the psychosocial outcome, these issues need to be investigated prospectively. Finally, multicenter studies with a larger number of participants can help rule out potential effects by the clinics' individual surgeon(s) and provide more robust results. Finally, some of the questionnaires used in the present study are not validated for transgender and gender-diverse populations, which could potentially impair the understanding of the results (see supplementary material for detailed discussion of this issue).

As the first of its kind, the present study introduced (de-)centralized health care delivery into transgender health care research. Therefore, our work should be viewed as an initial attempt to investigate genital gender-affirming procedures within a more comprehensive framework, including the various potential factors influencing the outcome of these interventions. However, this approach needs to be used in a study design with higher methodological quality and should be evaluated and reviewed repeatedly. To generate these high-quality data, a prospective study using the present theoretical framework and methodology is currently in progress.

## Supplementary Information

Below is the link to the electronic supplementary material.

Supplementary file1 (PDF 132 KB)

Supplementary file2 (PDF 201 KB)

Supplementary file3 (PDF 225 KB)

Supplementary file4 (DOC 86 KB)

## Data Availability

We will consider sharing de-Identified individual participant level data that underlie the results reported in this Article on receipt of a request detailing the study hypothesis and statistical analysis plan. All requests should be sent to the corresponding author. The corresponding author and lead investigators of this study will discuss all requests and make decisions about whether data sharing is appropriate based on the scientific rigour of the proposal. All applicants will be asked to sign a data access agreement.
