# Supplementary material for: (De-)centralized health care delivery, surgical outcome, and psychosocial health of transgender and gender-diverse people undergoing vaginoplasty: results of a retrospective, single-center study
Source: World J Urol. 2023 Mar 24;41(7):1775–83. doi: 10.1007/s00345-023-04348-5 (PMC10352146; doi:10.1007/s00345-023-04348-5)
Supplement: Supplementary file 1 — Supplementary file1 (PDF 132 KB) [file 345_2023_4348_MOESM1_ESM.pdf]

Discussion of univariate analyses of the outcomes

Besides the multivariate relationships found between the outcomes and predictors used in the present study, a discussion that explores each variable in our data set separately seems reasonable, as the sample size of the present study was small.

We found that the aesthetic outcomes after vaginoplasty was comparable to satisfaction in cisgender samples [1]. Moreover, participants from a centralized and a decentralized setting of healthcare delivery did not differ from each other. With a mean of 22.7 (centralized) and 22.0 (decentralized) on a scale of 28, the overall aesthetic satisfaction was high (Table S2). Vaginoplasty is a common genital gender-affirming procedure[2, 3], and surgeons trained in the appropriate surgical techniques can meet the expectation for postsurgical looks and appearance.

Contrary to our results concerning aesthetics, the functional outcomes were poor for both groups. They reported a mean of 11.3 (centralized) and 14.5 (decentralized) on a scale with a maximum of 36. However, as 44% of the sample reported not having sex within the last four weeks, they were considered as having a poor functional outcome. However, more than 40% of the participants in both groups also reported not being in a relationship and might have a lower likelihood of being sexually active with one or more other people. Also, 17-24% of the participants in both groups reported insufficient vaginal depth for penetrative intercourse. Even though sexual activity can and should not be reduced to penetrative intercourse, this might have favored the low scoring regarding the functional outcome of the procedure. Overall, these results reflect the existing challenges of genital gender-affirming procedures [2, 3] and show that, despite satisfying aesthetic outcomes, problems with sexual function after vaginoplasty need to be considered and adequately addressed by healthcare providers. Problems regarding urinary functioning were less common compared to sexual function problems. Only two participants from the centralized and three from the decentralized group reported experiencing those, which is lower than reported in systematic reviews of prior research[4]. The participants reported problems with a strong urinary streams and recurrent UTIs. Overall, it appears that limitations in sexual functioning are the major postoperative challenge after vaginoplasty. Also, these problems occurred in both the centralized and decentralized groups and might, therefore, not be influenced by the setting of health care delivery.

Univariate data analysis regarding gender congruence, psychological distress, and quality of life has also been done and will be reported in another manuscript [5].

The problem of using measures that are not validated in transgender and gender-diverse samples

Currently, there is a lack of quality, validated questionnaires to assess outcomes of genital gender-affirming surgery in transgender and gender-diverse populations. Most questionnaires are only validated in cisgender populations. This is also true for the FGSIS [1] and FSFI [6] used in the present study. Using non-validated instruments can potentially lead to inaccurate or unreliable results, which can impair the understanding of the outcomes of the procedure researched in a study. The lack of validated questionnaires for genital gender-affirming surgery in transgender and gender-diverse populations can also lead to inadequate standardization in assessing and reporting outcomes across studies. This can make it difficult to compare results and draw conclusions about the effectiveness of procedures. Moreover, there is a risk of bias, as questionnaires which are not validated may be susceptible to cultural bias. Therefore, they may not be suitable for all cultural backgrounds, which might lead to inaccurate assessment of individual participants.

Overall, the lack of validated questionnaires for genital gender-affirming surgery in transgender and gender-diverse populations is an important issue that needs to be addressed to improve the understanding of the outcomes of procedures and to facilitate the comparison of results across studies.

References

1. Herbenick D, Schick V, Reece M, Sanders S, Dodge B, Fortenberry JD. The Female Genital Self-Image Scale (FGSIS): Results from a nationally representative probability sample of women in the United States. The journal of sexual medicine. 2011;8(1):158-66.
2. Safer JD, Tangpricha V. Care of transgender persons. New England Journal of Medicine. 2019;381(25):2451-60.
3. Djordjevic ML, Bencic M, Kojovic V, Stojanovic B, Bizic M, Kojic S, et al. Musculocutaneous latissimus dorsi flap for phalloplasty in female to male gender affirmation surgery. World Journal of Urology. 2019;37(4):631-7.
4. Dreher PC, Edwards D, Hager S, Dennis M, Belkoff A, Mora J, et al. Complications of the neovagina in male-to-female transgender surgery: A systematic review and meta-analysis with discussion of management. Clinical Anatomy. 2018;31(2):191-9.
5. Koehler A, Strauß B, Briken P, Fisch M, Riechardt S, Nieder T. (De)centralized health care delivery, client-centeredness, and health outcomes in transgender and gender-diverse people undergoing vaginoplasty– Results of a retrospective, single-center study. under review.
6. Rosen CB, J. Heiman, S. Leiblum, C. Meston, R. Shabsigh, D. Ferguson, R. D'Agostino, R. The Female Sexual Function Index (FSFI): a multidimensional self-report instrument for the assessment of female sexual function. Journal of sex & marital therapy. 2000;26(2):191-208.
