# Supplementary material for: (De-)centralized health care delivery, surgical outcome, and psychosocial health of transgender and gender-diverse people undergoing vaginoplasty: results of a retrospective, single-center study
Source: World J Urol. 2023 Mar 24;41(7):1775–83. doi: 10.1007/s00345-023-04348-5 (PMC10352146; doi:10.1007/s00345-023-04348-5)
Supplement: Supplementary file 2 — Supplementary file2 (PDF 201 KB) [file 345_2023_4348_MOESM2_ESM.pdf]

Methods

Study design

The present retrospective study was conducted by the Institute for Sex Research, Sexual Medicine and Forensic Psychiatry and the Department for Urology, both located at the University Medical Center Hamburg-Eppendorf (UKE), Germany. Both are part of the Interdisciplinary Transgender Health Care Center Hamburg. It was performed of a single surgeon´s experience (SR) and was part of a larger research project on client-centered health care for transgender and gender-diverse people in Germany[19]. The study received ethical approval from the Chamber of Psychotherapists Hamburg Ethics Committee (10/2018-PTK-HH).

Participants

Participants had to be at least 16 years of age and underwent a two-step vaginoplasty using penile inversion technique to be eligible for study participation. All former patients who completed a vaginoplasty between 2014 and 2018 were invited to participate.

Participant recruitment

The data collection took place between January and March 2020. We identified all eligible former patients and invited them to participate in the study by letter. The invitation letter contained information on the study and the weblink to the online survey. If participants did not want to fill out the survey online, we offered to participate by mail or answer the survey on a desktop computer at the UKE. Participants had to give their informed consent before answering the survey. Of the 119 eligible former patients, three could not be traced. The remaining 116 former patients were contacted and asked to participate in the study. 45 former patients responded to our inquiry and were included in the study (response rate of 38.8 %). All participants answered at least 90% of the survey questions. We were not able to determine why former patients did not want to participate in the study. To assess a systematic bias in the recruitment procedure, a non-responder analysis was performed by comparing age and the size of place of residence between participants and non-participants, as these were the only data available from both groups. The size of place of residence from non-participants was determined by the postal address, whereas participants answered a question concerning this issue. Due to the method of participant recruitment, access to a web-enabled device and technical affinity need to be considered as potential biases.

Measures

The present analysis investigated data on the aesthetic and functional outcome of vaginoplasty, satisfaction with the treatment, gender congruence, mental health, and quality of life. When participants received counseling and maybe other treatments within the Interdisciplinary Transgender Health Care Center Hamburg, they were considered receiving care in a centralized healthcare delivery setting. Those who only underwent vaginoplasty at the Department for Urology were categorized as accessing transgender health care decentralized (Figure 1)[19]. We used the following standardized measures, all of which are PROMs:

1. *Transgender Congruence Scale[20]*
   The TCS is a 12-item questionnaire measuring the degree of comfort transgender and gender-diverse people feel with their gender and external appearance. The items are scored on a 5-point scale. For the overall score, item responses are averaged, with higher scores indicating higher levels of congruence.

2. *Brief Symptom Inventory-18 (BSI-18)[21]*

The BSI-18 is an 18-item self-report checklist measure to assess psychological symptoms. Items are rated on a 5-point scale. Scores are summarized on the Global Severity Index (GSI) and three subscales (somatization, depression, anxiety). The BSI-18 has been widely used in a variety of queer populations[22-24].

3. *WHOQOL-BREF[25]*
The WHOQOL-BREF measures quality of life on four dimensions (physical health, psychological health, social relationships, environment) using 26 items. The items are scored on a 5-point scale. The domain scores are transformed and scaled from 0 to 100.

4. *Female Genital Self Image Scale (FGSIS)[26]*
The FGSIS measures women´s genital self-image with seven questions on a 4-point scale. The maximum overall score is, therefore, 28. Higher scores indicate a better genital self-image.

5. *Female Sexual Functioning Index (FSFI)[27]*
The FSFI measures female sexual functioning on six subscales (desire, arousal, lubrification, orgasm, satisfaction, pain) in a total of 19 questions with a score of a maximum of 6 each. Therefore, the maximum overall score is 36. Higher scores indicate a better outcome regarding sexual functioning. A score of 26.55 is reported as the cut-off point for the existence of a sexual dysfunction[28].

6. *Adapted Short Questionnaire for Self-Evaluation of Vaginoplasty (SQSV)[29]*
The SQSV was used by Buncamper et al.[29] to assess both the functional and aesthetic outcomes of vaginoplasty. Initially, the authors only used dichotomous response options (yes, no). We adapted the questionnaire to our sample by introducing continuous response options (e.g., concerning satisfaction with the procedure). As other questionnaires we used already covered some issues addressed in the SQSV, we only used single items, not the whole questionnaire, as used by Buncamper et al.[29].

Data Analyses

The statistical analyses were conducted using SPSS 24.0. Missing data were deleted pairwise. The sample characteristics and outcomes of the questionnaires were reported descriptively (Tables 1 - 6). To illustrate the progress in transgender-related treatment, the Individuals Treatment Progress Score[1] was calculated. The Mann-Whitney U-test was calculated to assess differences between participants accessing transgender health care in centralized and decentralized delivery settings. We calculated hierarchical linear regression analyses to assess the relationship between psychosocial outcome measures (gender congruence, mental health, quality of life) and different aspects of gender-affirming genital surgery (treatment satisfaction, aesthetic outcome, functional outcome, setting of health care delivery). For gender congruence, the overall score of the GCS questionnaire was used as the dependent variable[20]; for psychological distress, the *Global Severity Index* of the BSI-18[21] was used as the dependent variable. Regarding the quality of life, the four sub-scales of the WHOQOL-BREF (physical health, psychological, social relationships, environment)[25] were used as dependent variables. A single item from the SQSV[29] concerning satisfaction with the treatment was used as a predictor for treatment satisfaction. The score of the FGSIS[26] was used as a predictor for the aesthetic outcome. The FSFI score[27] and a single item from the SQSV[29] concerning problems with urinary excretion were used as predictors for functional outcomes. The grouping variable of (de-)centralized delivery of care was used as a predictor for the setting of service delivery. The item on treatment satisfaction from the SQSV[29] was entered into step 1. The FGSIS-score[26] as a measure for the aesthetic outcome was entered into step 2. The FSFI-score[27] and the item on

problems with urinary excretion as measures for the functional outcome were entered into Step 3. The item on (de-)centralized delivery of health care was entered into step 4.
Normal distribution of data and residuals were examined using histograms and Q-Q plots. The assumption of the independence of the observations was assessed using the Durbin-Watson statistic. Multicollinearity was excluded by inspecting correlation coefficients and tolerance/ VIF (Variance inflation factor) values. Heteroscedasticity was assessed by examining scatterplots of predicted residuals. Using G*Power[30, 31], we calculated the minimum sample size necessary to find a significant effect a priori. Due to the lack of high-quality evidence[14-18], we assumed a large effect of our predictors on the psychosocial outcome based on clinical experience. Therefore, multiple regression analysis with five predictors and a power of .80 needs a sample size of at least N=43 to determine a large effect (f^2 = .35). We used Cohen´s f^2 as a post-hoc measure of the effect size of the regression models. According to Cohen´s conventions, f^2 = .02 indicates a small effect, f^2 = .15 indicates a medium effect, and f^2 = .35 indicates a large effect[32]. We also used G*Power[30, 31] to perform a post hoc calculation of the achieved power of our models. For the non-responder analysis, a t-test for independent samples and a chi-square test were performed to compare the age and population of the place of residence between participants and non-participants. All analyses were performed with an alpha level of .05, and – to handle the problem of multiple comparisons – a Bonferroni-corrected[33] alpha level of 0.01 (0.05 divided by the 5 predictors of the hierarchical regression analyses[34]).
