# Supplementary material for: (De-)centralized health care delivery, surgical outcome, and psychosocial health of transgender and gender-diverse people undergoing vaginoplasty: results of a retrospective, single-center study
Source: World J Urol. 2023 Mar 24;41(7):1775–83. doi: 10.1007/s00345-023-04348-5 (PMC10352146; doi:10.1007/s00345-023-04348-5)
Supplement: Supplementary file 3 — Supplementary file3 (PDF 225 KB) [file 345_2023_4348_MOESM3_ESM.pdf]

Table S1 *Gender- and treatment-related characteristics*

**Gender**	**N (%)**	
**Woman/female**	39 (88.6)	
**Transwoman**	14 (31.8)	
**Trans***	4 (9.1)	
**Transgender**	6 (13.6)	
**Transsexual**	5 (11.4)	
**Genderfluid**	1 (2.3)	
**Other**	Androgynous	1 (2.3)
	Woman with transsexual background	1 (2.3)
	Non-binary/enby, femby, demiflux	1 (2.3)
	Transident	1 (2.3)
**Non-binary gender**		
**No**	41 (93.2)	
**Yes**	3 (6.8)	
**Treatment progress (ITPS*)**		
**Mean (SD)**	.78 (.18)	
**Range**	.38 – 1.00	

*The ITPS (Individual Treatment Progress Score) ranges between 0 and 1. A higher score indicates a more advanced treatment[1].

Table S2 *Aesthetic outcome (Female Genital Self Image Scale[26])*

	Total sample (Mean [SD], Mdn*)	Centralized health care delivery (Mean [SD], Mdn*)	Decentralized health care delivery (Mean [SD], Mdn*)	Statistics
I feel positively about my genitals	3.6 (.5), 4.00	3.7 (.5), 4.0	3.5 (.6), 4.0	
I am satisfied with the appearance of my genitals	3.4 (.8), 4.00	3.4 (.8), 4.0	3.3 (.8), 4.0	
I would feel comfortable letting a sexual partner look at my genitals	3.4 (.8), 4.00	3.3 (.8), 3.0	3.4 (.8), 4.0	
I think my genitals smell fine	2.8 (1.0), 3.00	2.8 (1.1), 3.0	2.8 (1.0), 3.0	
I think my genitals work the way they are supposed to work	3.1 (.8), 3.00	3.1 (.6), 3.0	3.1 (0.9), 3.0	
I feel comfortable letting a healthcare professional examine my genitals	3.4 (.6), 3.00	3.6 (.5), 4.0	3.3 (.6), 3.0	
I am not embarrassed about my genitals	3.5 (.8), 4.00	3.5 (.9), 4.0	3.5 (.7), 4.0	
**Overall score**	22.0 (4.3), 23.0	22.7 (3.6), 23.0 (mean rank=22.07)	22.0 (5.2), 23.0 (mean rank=22.98)	U= 231.500; p=.813

*The FGSIS was answered on a 4-point scale. The maximum overall score is therefore 28. Higher scores indicate a better genital self-image.

Table S3 *Sexual function outcome (Female Sexual Function Index[27]) **

Subscale	Total sample (Mean [SD], Mdn)	Centralized health service delivery (Mean [SD], Mdn)	Decentralized health service delivery (Mean [SD], Mdn)	Statistics
Desire	2.8 (1.6), 2.4	2.8 (1.5), 2.4	2.7 (1.8), 1.8	
Arousal	2.7 (2.5), 2.7	2.5 (2.5), 1.4	3.0 (2.5), 3.0	
Lubrification	2.0 (1.6), 3.0	1.9 (1.6), 3.0	2.1 (1.7), 3.0	
Orgasm	2.7 (2.0), 3.6	2.9 (1.8), 3.8	2.5 (2.1), 3.2	
Satisfaction	2.4 (1.9), 1.6	2.0 (2.0), 1.2	2.8 (1.8), 2.4	
Pain	1.2 (1.5), 0.8	0.9 (1.3), 0.0	1.5 (1.7), 1.2	
**Overall Score**	12.8 (6.4), 13.9	11.3 (8.1), 10.9 (mean rank= 21.29)	14.5 (9.7), 17.1 (mean rank=24.95)	U= 211.000; p=.351

*Subscales could score a maximum of 6 each. The maximum overall score is 36. Higher scores indicate a better outcome. A total score<26.55 may be classified as having sexual dysfunctions

TableS4 *Aesthetic and functional outcome (modified version of the SQSV[29])*

	Total sample (Mean [SD], Mdn, no.)	Centralized health service delivery (Mean [SD], Mdn)	Decentralized health service delivery (Mean [SD], Mdn)	Statistics
**Satisfaction with result***	2.2 (1.3), 2.0	2.3 (1.5), 2.0 (mean rank= 22.46)	2.0 (1.0), 2.0 (mean rank= 22.55)	U= 240.500; p=.980
**Vaginoplasty outcome as expected?†**	1.8 (.9), 2.0	1.7 (0.8), 1.0 (mean rank= 20.57)	2.0 (0.9), 2.0 (mean rank= 24.62)	U= 197.000; p=.259
**Vagina deep enough for penetrative sexual intercourse?**				
Yes	28 (62.2)	15 (62.5)	13 (61.9)	
No	9 (20.0)	4 (16.7)	5 (23.8)	
Cannot or do not wish to answer this question	8 (17.7)	4 (16.7)	3 (14.3)	
**Appearance feminine enough?**				
Yes	38 (84.4)	20 (83.3)	18 (85.7)	
No	5 (11.1)	3 (12.5)	2 (9.5)	
Cannot or do not wish to answer this question	2 (4.4)	1 (4.2)	1 (4.8)	
**Urinary problems**				
Yes (see table 6 for the individual answers)	5 (11.1)	2 (8.3)	3 (14.3)	
No	39 (86.6)	21 (87.5)	18 (85.7)	
Cannot or do not wish to answer this question	1 (2.2)	1 (4.2)	0 (0.0)	

*The item was scored between 1 (very satisfied) to 5 (very unsatisfied)

†The item was scored between 1 (totally) to 5 (not at all)

Table S5 *Free text responses on urinary problems after vaginoplasty*

**Urinary problem**	**N**
Mostly not a strong urinary stream, more like wetting of the butt cheeks	1
Weak urinary stream	1
Frequent urinary tract infections	1
Irregular pain and frequent urinary tract infections	1
Split urinary stream and difficulties	1
